# The Cas9-gRNA ribonucleoprotein complex-mediated editing of *pyrG* in *Ganoderma lucidum* and unexpected insertion of contaminated DNA fragments

**DOI:** 10.1038/s41598-023-38331-2

**Published:** 2023-07-10

**Authors:** Hyerang Eom, Yeon-Jae Choi, Rutuja Nandre, Hui-Gang Han, Sinil Kim, Minseek Kim, Youn-Lee Oh, Takehito Nakazawa, Yoichi Honda, Hyeon-Su Ro

**Affiliations:** 1 Department of Bio&Medical Bigdata (BK21) and Research Institute of Life Sciences, Gyeongsang National University, Jinju, 52828 Republic of Korea; 2 Mushroom Science Division, National Institute of Horticultural and Herbal Science, Rural Development Administration, Eumseong, 27709 Republic of Korea; 3 Laboratory of Forest Biochemistry, Graduate School of Agriculture, Kyoto University, Kyoto, 606-8502 Japan

**Keywords:** Fungal genetics, Genetic transduction

## Abstract

Gene editing is a promising alternative to traditional breeding for the generation of new mushroom strains. However, the current approach frequently uses Cas9-plasmid DNA to facilitate mushroom gene editing, which can leave residual foreign DNA in the chromosomal DNA raising concerns regarding genetically modified organisms. In this study, we successfully edited *pyrG* of *Ganoderma lucidum* using a preassembled Cas9-gRNA ribonucleoprotein complex, which primarily induced a double-strand break (DSB) at the fourth position prior to the protospacer adjacent motif. Of the 66 edited transformants, 42 had deletions ranging from a single base to large deletions of up to 796 bp, with 30 being a single base deletion. Interestingly, the remaining 24 contained inserted sequences with variable sizes at the DSB site that originated from the fragmented host mitochondrial DNA, *E. coli* chromosomal DNA, and the Cas9 expression vector DNA. The latter two were thought to be contaminated DNAs that were not removed during the purification process of the Cas9 protein. Despite this unexpected finding, the study demonstrated that editing *G. lucidum* genes using the Cas9-gRNA complex is achievable with comparable efficiency to the plasmid-mediated editing system.

## Introduction

Mushrooms, which are fungal species that mostly belong to Basidiomycota, are widely distributed organisms that play a critical role in carbon cycling through their decomposition activities. Some mushroom species, such as *Agaricus bisporus*, *Pleurotus ostreatus*, *Lentinula edodes*, and *Ganoderma lucidum*, have been utilized for both medicinal and culinary purposes in human society for a considerable time. These species have become significant agricultural commodities globally, and consequently, new cultivars are continually needed to enhance quality and productivity.

Mushroom cultivars have been primarily generated by conventional cross-mating of haploid mycelial strains of different genetic backgrounds. However, it is a time-consuming process that requires human efforts and financial inputs because obtaining a desirable strain is difficult. Molecular breeding, which involves altering genes of interest in a targeted manner, has been sought to precisely modify some mushroom strains in recent decades^1–3^. However, this technique is not feasible for many mushrooms due to the lack of proper molecular biological tools^4^. In contrast to prokaryotes and yeasts, mushrooms do not have autonomously replicative cytoplasmic plasmids, which makes it difficult to introduce foreign genes into mycelial cells. Integrating foreign DNA fragments into chromosomal DNA is also difficult because nonhomologous end-joining (NHEJ) is the main mechanism for DNA damage repair, which instantly repairs double strand breaks (DSBs) and therefore rarely allows homologous recombination (HR)^5,6^. Forced chromosomal integration of foreign DNA and selection under antifungal agents or auxotrophic markers have generally been used for the transformation of mushrooms, delivered by *Agrobacterium tumefaciens*-mediated transformation (ATMT)^3,7–9^ or PEG-mediated transformation^1,2^. However, the method is not applicable in commercial strain development due to issues with genetically modified organisms (GMOs).

Gene editing in mushrooms has emerged as a promising molecular breeding tool to perform targeted manipulation of genes related to specific traits. The announcement of the gene-edited *A. bisporus* to bypass US GMO regulations in 2016 has been a significant driver of this approach^10^. Several species of mushrooms, including *Ceriporiopsis subvermispora*^11^, *Coprinopsis cinerea*^12^, *Cordyceps militaris*^13–15^, *Flammulina filiformis*^16^, *G. lucidum*^17–20^, *L. edodes*^21^*, P. ostreatus*^22–24^, and *P. eryngii*^25^, have been subjected to gene editing through the introduction of plasmid DNA carrying Cas9 and guide RNA (gRNA). The plasmid DNA here is either integrated into the chromosomal DNA or diluted out along with mycelial cell division due to the absence of autonomously replicative sequences. ATMT is another way to introduce the Cas9 and gRNA genes. Zhang et al.^26^ achieved the disruption of *pyrG* by chromosome-integrated Cas9 and gRNA and subsequent disruption of *lacA* or *mnp9* through additional integration of gRNA targeting *lacA* or *mnp9* using ATMT.

Another intriguing approach to implementing gene editing is by directly introducing the Cas9-gRNA ribonucleoprotein complex (RNP) into protoplasts. The major advantage of this approach is that it does not involve chromosomal integration of Cas9/gRNA, enabling marker-free editing. In Vonk et al.’s^27^ study, RNP was utilized to create a targeted DSB in the *hom2* gene of *Schizophyllum commune*. Selection of the *hom2*-edited cells was facilitated by cotransformation of a DNA fragment containing a selective marker gene flanked by homologous sequences of *hom2*, which was integrated into the *hom2* gene through HR upon the creation of a DSB. A similar strategy was employed to edit *cre1* of *Coprinopsis cinerea*^28^ and *pyrG* of *P. ostreatus*^29^.

*G. lucidum* is commonly found in Asia^30^ and has gained popularity due to its active compounds, such as ganoderic acids, which have been demonstrated to effectively treat various diseases, such as cancer, diabetes, and hypertension; as a result, *G. lucidum* is a valuable source of medicinal substances^31–33^. Qin et al.^17^ reported the first gene-edited *G. lucidum* by plasmid carrying the Cas9 gene with in vitro transcribed gRNA targeting *pyrG* (*ura3*), which encodes orotidine 5-phosphate decarboxylase and has been a frequent target in gene editing due to its disruption enables positive selection against 5-fluoroorotic acid (5-FOA). The *pyrG*-edited strain was further subjected to modification of *cyp5150l8,* which is involved in the biosynthesis of ganoderic acids^18^. Similar plasmid-mediated editing was performed to target *pyrG* and *GL17624*^19^ and *ku80* and *pyrG*^20^. These plasmid-based editing systems have their own issues in general use, in which several factors should be considered, such as codon optimization of the Cas9 gene, promoter selection for the expression of Cas9 and gRNA, use of selective markers, and integration of plasmid DNA fragments into chromosomal DNA.

In this regard, we attempted to develop a gene editing system in *G. lucidum* using purified Cas9 protein assembled together with in vitro transcribed gRNA. We found that the Cas9-gRNA RNP could effectively edit *pyrG* with a broad range of deletions through NHEJ repair. We also found that the DSB was repaired by insertion of random DNA fragments, originating from the transformation reaction mixture and from the mitochondrial DNA (mtDNA) fragments.

## Results

**Figure 1:**
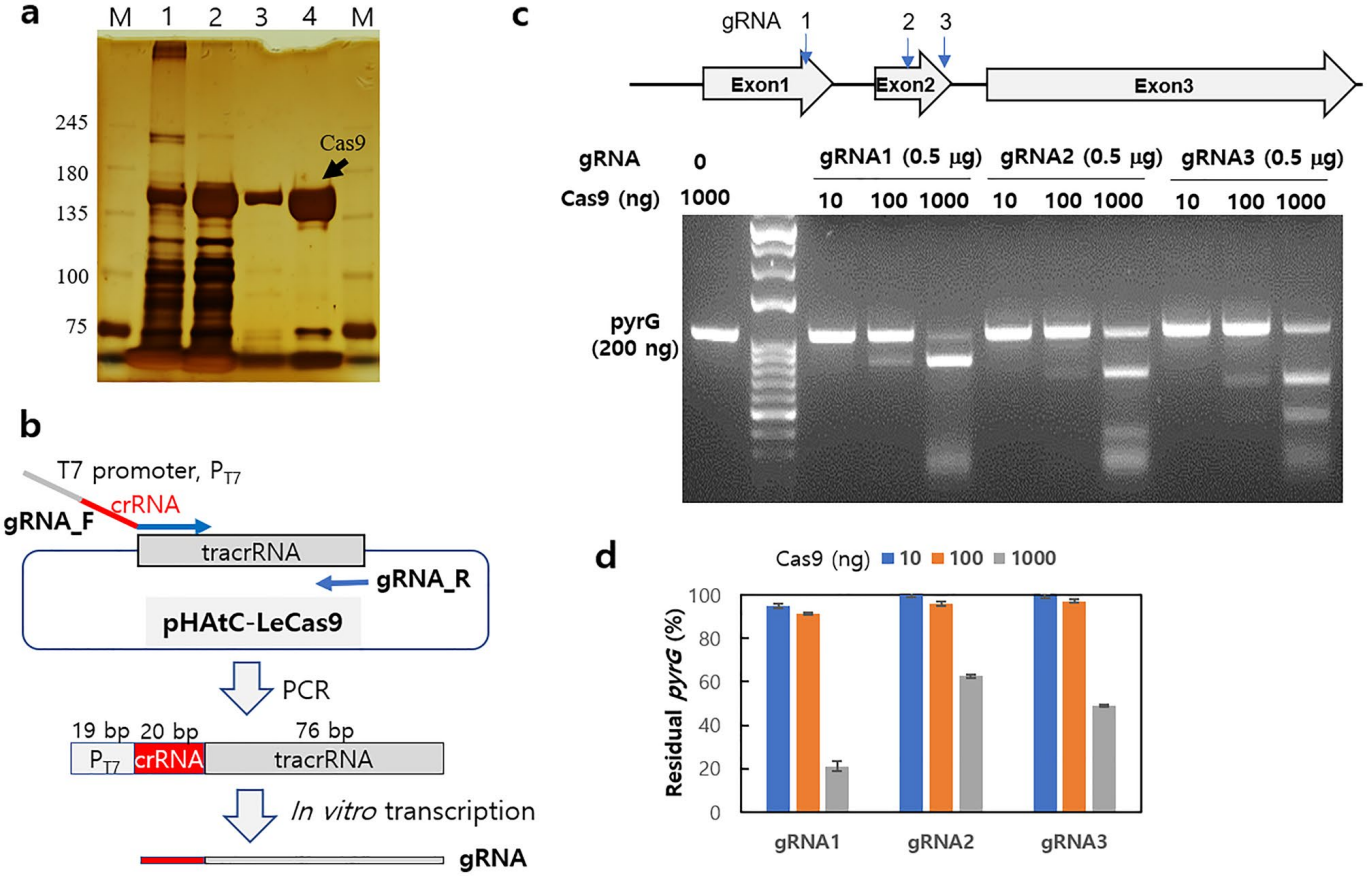
Preparation of Cas9 and in vitro cleavage assay. (**a**) Purification of the Cas9 protein. Lane 1: *E. coli* cell lysate. Lane 2: Column-bound proteins. Lane 3: Proteins eluted during washing with 20 mM imidazole. Lane 4: Eluted fraction with 1 M imidazole. Lane M: Molecular weight marker in kDa. (**b**) Scheme for the synthesis of gRNA. (**c**) In vitro cleavage assay by the Cas9-gRNA complex. The cleavage sites by gRNAs are indicated by arrows. Purified Cas9 in different amounts (10, 100 and 1000 ng) was incubated together with 0.5 μg gRNA and PCR-amplified *pyrG* (200 ng) at 37 °C for 10 min. (**d**) The cleavage efficiency of the Cas9-gRNA complex. The *pyrG* DNA intensities at different concentrations of Cas9 were measured by ImageJ, and the relative amount of residual *pyrG* was compared with the *pyrG* input. The data are the average values of triplicate measurements.

The Cas9 protein expressed in *E. coli* was purified by Ni–NTA column chromatography (Fig. 1a). SDS‒PAGE analysis of the eluted fraction (Fig. 1a, Lane 4) showed that Cas9 was the major protein, while minor contaminants with molecular weights of less than 75 kDa were also present. These minor proteins did not affect the Cas9 cleavage activity (Fig. 1c) and were thought to be *E. coli* proteins non-specifically bound to Ni–NTA column during the purification process. The gRNA was synthesized by in vitro transcription as depicted in Fig. 1b. Next, we examined cleavage of *pyrG* by gRNA-guided Cas9 activity. The *pyrG* DNA (1065 bp) was cut in a Cas9 protein concentration-dependent manner (Fig. 1c). However, the cleavage efficiency varied depending on the gRNA used. For example, Cas9 with gRNA1, which targets the first exon in *pyrG*, cut most of the target sequence into two fragments with sizes of 826 bp and 239 bp. *pyrG* band density analysis revealed that Cas9 complexed with gRNA1, gRNA2, and gRNA3 cleaved 79%, 37%, and 51% of input *pyrG* DNA, respectively (Fig. 1d).

**Figure 2:**
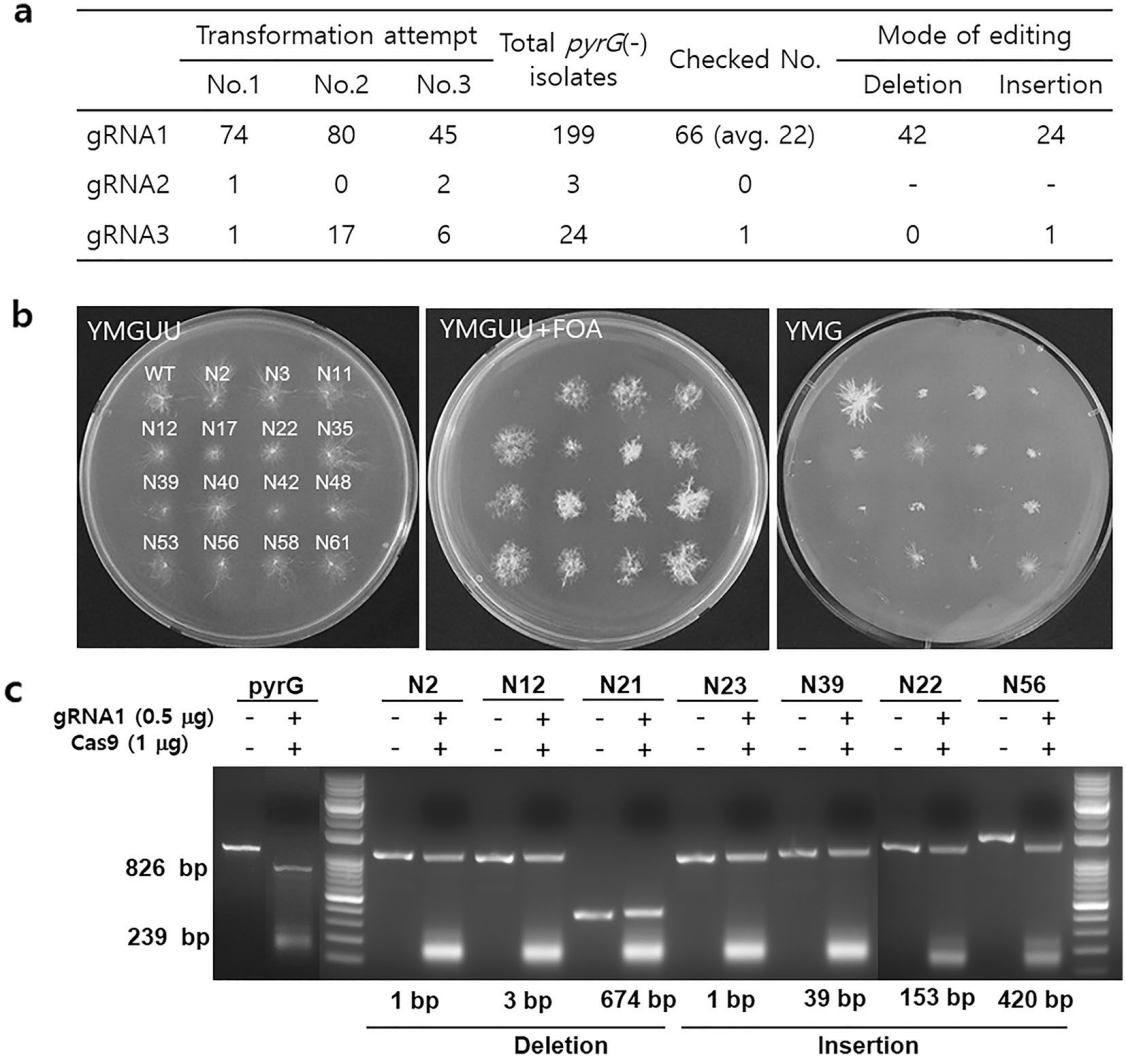
Isolation of *pyrG*-edited transformants. (**a**) Summarized results of PEG-mediated transformation with Cas9-gRNAs. Checked Nos. are the numbers of *pyrG*(–) isolates subjected to sequence analysis. (**b**) Growth characteristics of selected transformants. The transformants showed normal growth on YMGUU. The transformants grew in the presence of 5-FOA (YMGUU + FOA), while the wild type could not. The wild-type grew well on YMG, which lacked uracil and uridine, whereas the transformants rarely grew because of the disrupted pyrG. (**c**) Investigation of the *pyrG*s from the transformants. An in vitro cleavage assay by the Cas9-gRNA1 complex was performed on *pyrG*s from the selected transformants. The wild-type pyrG was cut into two pieces, while the edited pyrG remained intact. Sequence analysis revealed that they carried deletions or insertions.

To investigate the effect of gRNA on the transformation efficiency, the three Cas9-gRNA complexes were assembled by incubating the purified Cas9 protein with the in vitro-synthesized gRNA1, gRNA2, or gRNA3. The Cas9-gRNA complex was independently introduced to the protoplasts by PEG-mediated transformation. The transformants were selected based on their ability to survive or die on YMGUU + FOA medium. Only transformants with disrupted *pyrG* could survive in the presence of 5-FOA, as the orotidine 5'-phosphate (OMP) decarboxylase encoded by *pyrG* converts 5-FOA into lethal 5-fluorouracil. After conducting three independent transformation experiments, we retrieved a total of 199, 3, and 24 isolates from gRNA1, gRNA2, and gRNA3, respectively (Fig. 2a). The fast-growing isolates were further selected on a new YMGUU + FOA to obtain 66 transformants from gRNA1 and one from gRNA3. The growth characteristics of some selected transformants are shown in Fig. 2b, in which the transformants grew well on YMGUU + FOA, while the wild-type strain could not. The transformants were less viable on YMG medium, which lacked uracil and uridine, because of the disrupted *pyrG*.

**Figure 3:**
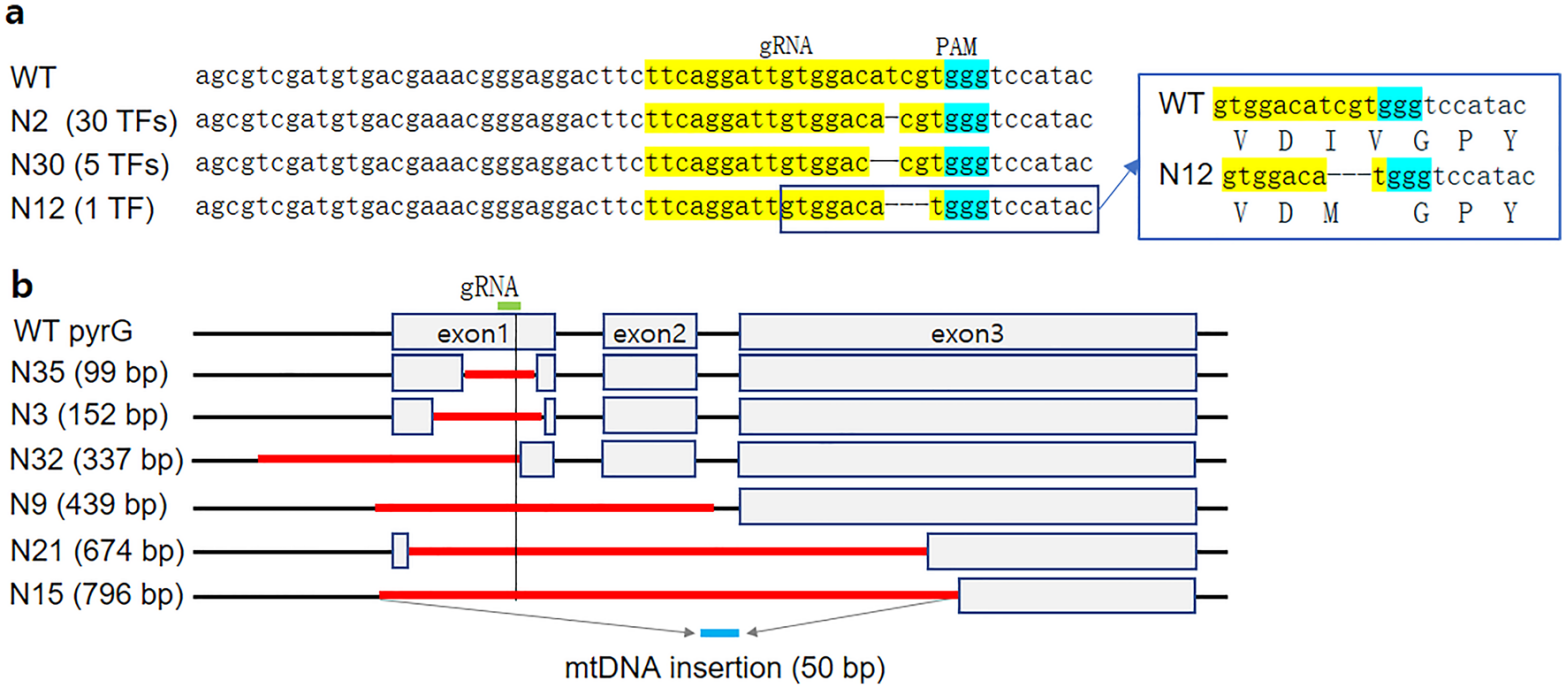
Transformants carrying deleted sequences in *pyrG*. (**a**) Transformants showing simple sequence deletion. The 36 transformants (TFs) showed single base (30 transformants), two base (5 transformants), or three base (1 transformant) deletions. The three base-deleted pyrG (N12) was functionally inactive due to the change in the amino acid residues isoleucine 52 and valine 53 into methionine 52 (boxed). The gRNA (crRNA) regions are shaded in yellow, while the PAM sequence is shown in cyan. (**b**) Transformants showing large deletions. The gRNA region is depicted as a green bar. The thick red line on the sequence indicates the deleted sequence region. The mitochondrial DNA replacing the 796 bp-deleted sequence in N15 is depicted as a blue line. The deleted sequences are shown in Supplementary Fig. S1.

**Figure 4:**
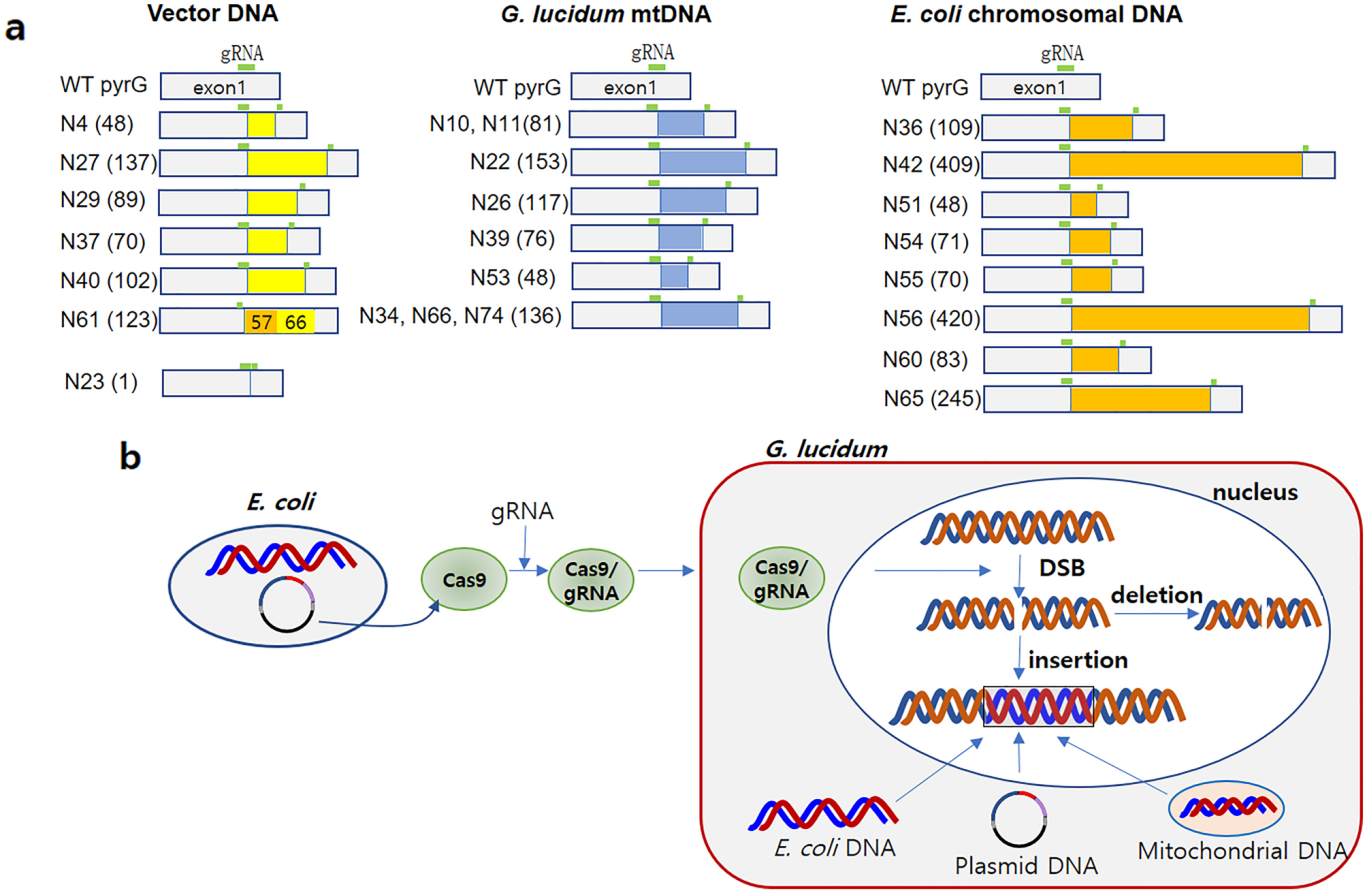
Edited *pyrG* carrying inserted sequences. (**a**) The inserted sequences originating from the pTrc99-Cas vector, *G. lucidum* mtDNA, and *E. coli* chromosomal DNA are yellow-, blue-, and orange-boxed, respectively. The inserted sequences are summarized in Supplementary Table S2. (**b**) Graphical description of the origin of the inserted DNAs. The *E. coli* chromosomal DNA and the vector DNA fragments generated during the preparation of the Cas9 protein enter the cytoplasm during transformation together with the purified Cas9 protein. The intracellular mtDNA fragments generated during the transformation process are inserted during NHEJ repair.

We subsequently investigated the *pyrG* gene in the transformants selected from gRNA1 using the in vitro cleavage assay. The *pyrG* DNA in the transformants was amplified by PCR and reacted with the Cas9 protein and gRNA1. While the wild-type *pyrG* was cut by two pieces through the activity of the Cas9-gRNA1 complex (Fig. 2c), all *pyrG*s from the transformants were resistant to Cas9-gRNA1, indicating modifications in the sequence region corresponding to crRNA. Moreover, there were noticeable changes in the length of *pyrG* from the transformants N21, N39, N22, and N56. *pyrG* from N21 was reduced to 674 bp, whereas N39, N22, and N56 carried noticeably larger *pyrG* than that of the wild type (Fig. 2c). *pyrG*s in the 66 transformants were amplified by a primer set (Supplementary Table S1) and were subjected to sequence determination. Investigation of the obtained sequences revealed that all 66 *pyrG*s were modified by the Cas9-gRNA1 complex, which potentially led to the inactivation of OMP decarboxylase. Forty-two transformants were found to contain deletions in the *pyrG* region (Fig. 3), whereas twenty-four contained insertions with variable lengths and origins (Fig. 4a). Among the deletions, thirty were single base deletions at the fourth position before the PAM sequence (Fig. 3, N2 as a representative transformant). The next most frequent deletion was a two-base deletion at the fourth position before PAM, found in five transformants, including N30. The transformant N12 carried a three-base deletion at the second position before PAM, which caused amino acid residues isoleucine 52 and valine 53 to change into methionine 52 (Fig. 3a, boxed). This change effectively disrupted the OMP decarboxylase activity, as shown by survival on YMGUU + FOA and death on YMG (Fig. 2b). Large deletions were also found in the six transformants (Fig. 3b, Supplementary Fig. S1). The transformants N3 and N35 showed 152-bp and 99-bp deletions, respectively, in exon 1 of *pyrG*. The deleted regions included the gRNA1 target sequence. N32 showed a 337-bp deletion, ranging from the fourth position before PAM to the upstream 179-bp region. The N9 transformants showed a deletion of 439 bp, including the upstream 84 bp, exons 1 and 2, and a part of the second intron sequence. The second largest deletion was found in N21, in which a 674-bp deletion occurred from 29 bp after the start codon to a significant part of exon 3. N15 showed the largest deletion (796 bp), ranging from 92 bp upstream to half of exon 3. Interestingly, in this transformant, the deleted sequence was replaced by a 50-bp DNA fragment, which belonged to a noncoding sequence region of *G. lucidum* mtDNA.

Sequence analysis of *pyrG*s revealed that twenty-four transformants contained insertions of different sizes at the gRNA region of exon 1 (Fig. 4a, Supplementary Table S2). The insertions primarily occurred at the 4th base from the PAM sequence. The smallest insertion was a single base insertion in N23, whereas the largest was 420 bp found in N42. Detailed sequence analysis revealed that the inserted sequences come from the following different origins: pTrc99-Cas9 plasmid DNA, *E. coli* chromosomal DNA, and the mtDNA of *G. lucidum* (depicted in Fig. 4b). Six transformants, including N4, N27, N29, N37, N40, and N61, carried inserted sequences originating from the vector DNA pTrc99-Cas9, which was used to produce the Cas9 protein (Fig. 4a, Supplementary Fig. S2). DNA fragments from *E. coli,* which was the host for pTrc99-Cas9, were also found to be inserted into 8 transformants (Fig. 4a). These results indicate that the fragmented DNAs generated during the preparation of the Cas9 protein remained as contaminants in the protein solution. Then, these fragments were incorporated into the target sequence when the DSB occurred due to Cas9-gRNA1 activity. The other type of inserted sequence was from mtDNA of *G. lucidum* (Fig. 4a, Supplementary Fig. S2). Nine transformants carried mtDNA fragments, of which N10 and N11 had the same insertion, which originated from the *cox3* gene, and N34, N66, and N74 possessed the same inserted sequence, which originated from *cox1*. These mtDNA fragments conceivably originated from disrupted mitochondria during the transformation process. The repeated occurrence of the two mtDNA fragments in the five transformants (N10, N11, N34, N66, and N74) may suggest nonrandom fragmentation of mtDNA (Supplementary Fig. S2). In contrast, the inserted sequences were randomly distributed to the vector DNA and *E. coli* chromosomal DNA. The transformant generated from gRNA3 contained the inserted sequence originating from *E. coli* chromosomal DNA (Supplementary Table S2).

## Discussion

Over the past few years, significant advancements have been made in the field of mushroom gene editing, particularly through successful transformations using plasmid DNA carrying the cas9 gene into protoplasts. However, for the practical use of this plasmid-based editing system, various factors must be carefully considered. For example, the appropriate promoters for the expression of Cas9 and gRNA must be chosen, the codons of Cas9 must be optimized to ensure efficient translation into protein, which requires a selective marker for cytoplasmic maintenance, and fragmented plasmid DNA may be randomly integrated into chromosomal DNA. In this regard, directly introducing the Cas9-gRNA RNP complex to protoplasts has been attempted for gene editing in mushrooms, such as *S. commune*^27^, *C. cinerea*^28^, and *P. ostreatus*^29,34^.

In this study, we found that the preassembled RNP was effective in disrupting *pyrG* in *G. lucidum* by inducing a double-strand break at the 4th base before PAM, similar to the results obtained for *F. filiformis*^16^ and *P. ostreatus*^22^. The efficiency of transformation was found to be highly dependent on the gRNA used and was directly proportional to the in vitro cleavage activity of the Cas9-gRNA complex. Out of the three gRNAs tested, only gRNA1, which exhibited the best cleavage activity in the in vitro cleavage assay (Fig. 1c), consistently produced a reasonable number of transformants, averaging 22 per 10^7^ protoplasts (as shown in Fig. 2a). Compared to the plasmid method, which exhibited an average efficiency of *pyrG* disruption of 16 per 10^7^ protoplasts^19^, our RNP-based transformation was found to be comparable.

Disruption of the *pyrG* gene was observed to result from deletions and insertions around the DSB site. Out of the 42 deleted transformants, 30, 5, and 1 exhibited single base, two base, and three base deletions, respectively, while the remaining six transformants showed larger deletions ranging from 99 to 796 bp, which may have occurred during the repair process via NHEJ. A more intriguing observation from the repair process was the insertion of DNA fragments from various sources. Specifically, 24 transformants were found to contain inserted sequences ranging in size from a single base to 420 bp; based on sequence analysis, these sequences originated from the following sources: contaminated DNA fragments in the purified Cas9 protein and mtDNA of host cells. The DNA fragments contaminating the purified Cas9 proteins originated from the expression vector DNA and the *E. coli* chromosomal DNA generated during the disruption of the Cas9-overexpressing *E. coli* cells by sonication. Additionally, mtDNA fragments may have been generated during the transformation process. As mushrooms may contain a substantial amount of mtDNA (similar to yeast cells, which can have 50–200 mitochondria^35^), the high osmotic and physicochemical stresses during protoplast generation and PEG-mediated transformation could have accelerated mtDNA degradation by nuclease activities^36^, resulting in the accumulation and insertion of degraded mtDNA fragments. Notably, this study found that certain transformants contained identical insertions in N10 and N11 and in N34, N66, and N74 (Fig. 4a), which suggests the involvement of a specific endonuclease in mtDNA degradation. Lastly, the random insertion of DNA fragments into the DSB site suggests that insertion of a specific DNA fragment can be facilitated when the RNP is transformed together with a DNA fragment with a specific function without using HR. This type of RNP-mediated gene editing and subsequent positive selection was employed in the double-gene targeting of *P. ostreatus*, in which both *pyrG* and *fcy1* (encoding cytosine deaminase) were disrupted by a single transformation of Cas9/*pyrG*sg1 and Cas9/*fcy1*sg2 RNPs and selected against 5-FOA and 5-fluorocytosine (5-FC)^34^. Deamination of 5-FC by *fcy1* produces 5-FU which is the same toxic product produced by *pyrG* on 5-FOA.

Although the use of RNPs enables marker-free editing, it has limitations regarding the selection process for modified organisms. These limitations arise unless the disruption of the target gene results in a discernible phenotype, such as the survival of the edited organism under 5-FOA selection due to pyrG disruption. Therefore, RNP-mediated gene editing is constrained when it comes to targeting arbitrary genes unless an effective selection strategy is in place.

## Materials and Methods

### Strain and culture conditions

A monokaryotic strain of *G. lucidum* was isolated by dedikaryotization of a dikaryotic strain (GL3315), which was isolated from the Sorak Mountains located in the northwestern part of Korea. The isolated strain was cultured at 28 °C on YPD medium containing yeast extract (5 g/L) and potato dextrose agar (24 g/L) or on minimal medium composed of asparagine (2 g/L), MgSO_4_ (0.12 g/L), dextrose (5 g/L), potato starch (5 g/L), and thiamine (1 µg/L).

### Design and synthesis of gRNA

Three crRNAs targeting *pyrG* of *G. lucidum* (GenBank ID: JQ406674.1) were designed using CRISPOR (http://crispor.tefor.net/). For the synthesis of gRNA, the T7 promoter (P_T7_), crRNA, and tracrRNA were connected by PCR with primer sets (Supplementary Table S1) using pHAtC-LeCas9^21^ as template DNA. The resulting DNA fragments were subjected to in vitro transcription using an in vitro RNA synthesis kit (NEB, Ipswich, MA). The gRNAs were purified using an RNA purification kit (Invitrogen, Waltham, MA).

### Purification of Cas9 protein

The Cas9 expression vector (pTrc-Cas9) was obtained from Prof. Hojin Ryu at Chungbuk National University. *E. coli* carrying pTrc-Cas9 was grown in LB broth containing ampicillin (50 μg/mL) at 37 ℃. Expression of Cas9 was induced by 0.1 mM isopropyl β-d-1-thiogalactopyranoside (IPTG) when the OD600 reached 0.6. The induced broth was further incubated for 16 h. The cells were harvested by centrifugation at 10,000×*g* for 20 min. The collected cells were disrupted by sonication in a 50 mM sodium phosphate buffer containing 0.1 M NaCl (pH 7.5). The disrupted solution was centrifuged at 10,000×*g* for 30 min (4 °C). The supernatant was collected and applied to a Ni–NTA column (10 mL) equilibrated with 50 mM sodium phosphate buffer containing 0.1 M NaCl (pH 7.5). The column was washed with 100 ml of equilibration buffer supplemented with 20 mM imidazole. The bound Cas9 protein was eluted using 1 M imidazole in the equilibration buffer. The eluate was dialyzed against 50 mM sodium phosphate buffer for 12 h at 4 °C to obtain the purified Cas9 protein. A Bradford assay was performed to determine the protein concentration.

### Evaluating the gRNA performance by an in vitro cleavage assay

The efficiency of the gRNAs in the editing of *pyrG* was evaluated by an in vitro cleavage assay. Purified Cas9 at different concentrations (10, 100, and 1000 ng) was incubated with the three gRNAs (500 ng) in the presence of 200 ng *pyrG* DNA fragment, which was amplified by PCR using chromosomal DNA extracted from *G. lucidum*. The cleavage reaction was performed in reaction buffer (20 μL) consisting of 50 mM Tris–HCl, 0.1 M NaCl, 10 mM MgCl_2_, and 1 mM DTT (pH 7.9) at 37 °C for 10 min. The reaction mixture was analyzed by 1.5% agarose gel electrophoresis. The substrate *pyrG* DNA was amplified by PCR with a primer set (Supplementary Table S1) using the following conditions: initial denaturation at 95 °C for 5 min; 25 cycles of denaturation at 95 °C for 30 s, annealing at 57 °C for 30 s, and extension at 72 °C for 10 s; and final extension at 72 °C for 5 min. The cleavage efficiency was calculated by the ImageJ program (https://imagej.nih.gov/ij/download.html).

### Preparation of protoplasts

*G. lucidum* was grown in 200 ml of YPD for 3 d at 25 ℃. The mycelia were harvested by centrifugation at 3500×*g* for 20 min and then washed twice with sterile distilled water. The collected mycelia were treated with cell wall lysing enzyme solution containing 0.5 M sorbitol, 1% β-glucanase (Amicogen, Jinju, Korea), 0.4% chitinase (Chitimax-N; Amicogen, Jinju, Korea), and 1% cellulase (Sigma‒Aldrich, St. Louis, MO) for 4 h at 30 ℃ with gentle agitation. The resulting solution was filtered through Miracloth (Merck, Darmstadt, Germany) to remove undigested mycelial fragments. The filtrate was centrifuged at 1000×*g* for 8 min. The precipitant was washed with 1 ml of STC buffer (10 mM Tris–HCl, 10 mM CaCl_2_, and 1 M sorbitol, pH 7.4). The protoplasts were resuspended in 200 μL STC buffer for transformation. The number of protoplasts was counted under a microscope using a hemocytometer.

### Transformation of *G. lucidum* by the Cas9 and gRNA complex

The Cas9 protein and gRNA nucleoprotein complex was assembled by incubating 15 μg of the purified Cas9 protein and 7.5 μg of gRNA in 20 μL of H buffer (50 mM Tris–HCl, 0.1 M NaCl, 10 mM MgCl_2_, and 1 mM DTT, pH 7.9) at 37 °C for 30 min. The RNP solution was mixed with 200 μL of the protoplast solution (final 1.0 × 10^7^ protoplasts) and 2 μL of Triton X-100. The RNP-protoplast solution was incubated on ice for 40 min. After incubation, 1 ml of PTC buffer (40% PEG4000 in STC buffer) was added and incubated at 25 °C for 20 min. The mixture was spread on selection medium (YMGUU + FOA) consisting of yeast extract (4 g/L), malt extract (10 g/L), glucose (4 g/L), uracil (0.02 g/L), uridine (5 g/L), 5-FOA (1 g/L), and agar (15 g/L). The mycelial colonies grown out of YMGUU + FOA were transferred to new selection medium plates after 2 weeks of incubation at 28 ℃. The final isolates were transferred to YMGUU without 5-FOA for further analysis.

## Supplementary Information

Supplementary Legends.

Supplementary Figure S1.

Supplementary Figure S2.

Supplementary Information 1.

Supplementary Information 2.

Supplementary Information 3.

Supplementary Tables.

## Data Availability

All data generated or analysed during this study are included in this published article and its supplementary information files.
