# Supplementary material for: The Cas9-gRNA ribonucleoprotein complex-mediated editing of pyrG in Ganoderma lucidum and unexpected insertion of contaminated DNA fragments
Source: Sci Rep. 2023 Jul 10;13:11133. doi: 10.1038/s41598-023-38331-2 (PMC10333205; doi:10.1038/s41598-023-38331-2)
Supplement: Supplementary file 1 — Supplementary Legends. [file 41598_2023_38331_MOESM1_ESM.docx]

**Legends for Supplementary Figures**

**Supplementary Fig. S1. The deleted sequences in the *pyrG* gene.** The gRNA (crRNA) regions are shaded in yellow while the PAM sequence is in cyan. The start codon of pyrG is bold-faced with underline. The 50-bp mtDNA fragment inserted in N15 is red-faced.

**Supplementary Fig. S2. The origins of the inserted sequences.** The red arrows indicate the sequence that inserted into the pyrG-edited transformants.

**Supplementary gel image for Figure 1b**

**Supplementary gel image for Figure 2b**

**Supplementary gel image for Figure 2b_control**
