# Supplementary figures and images for: The Cas9-gRNA ribonucleoprotein complex-mediated editing of pyrG in Ganoderma lucidum and unexpected insertion of contaminated DNA fragments
Source: Sci Rep. 2023 Jul 10;13:11133. doi: 10.1038/s41598-023-38331-2 (PMC10333205; doi:10.1038/s41598-023-38331-2)

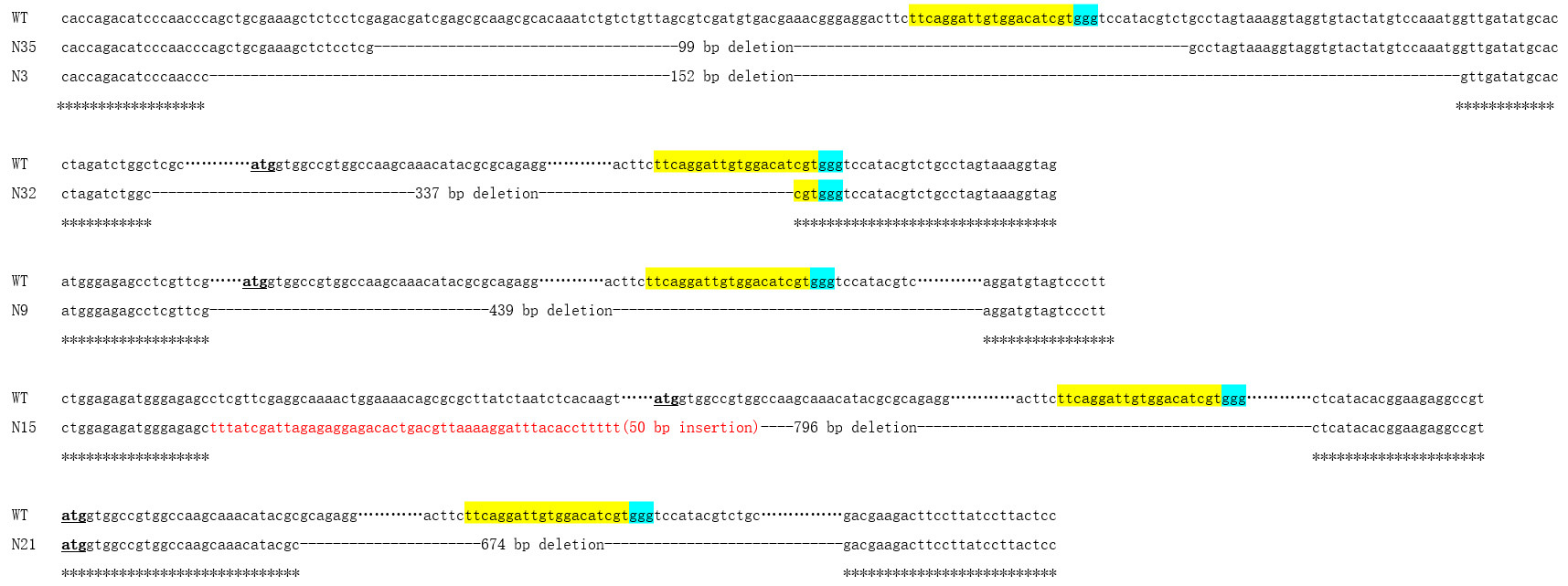

Supplement: Supplementary file 2 — Supplementary Figure S1. [file 41598_2023_38331_MOESM2_ESM.jpg]

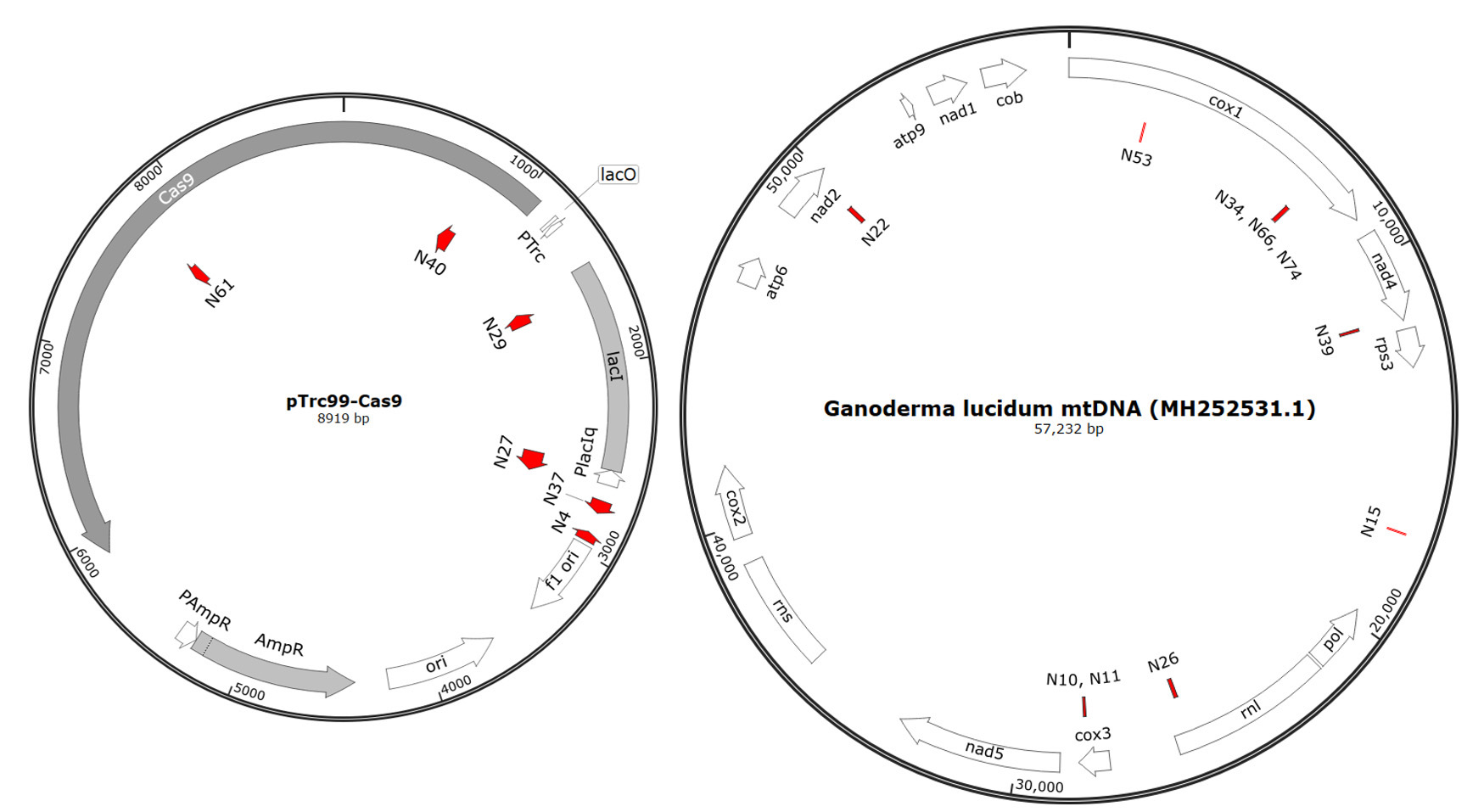

Supplement: Supplementary file 3 — Supplementary Figure S2. [file 41598_2023_38331_MOESM3_ESM.jpg]

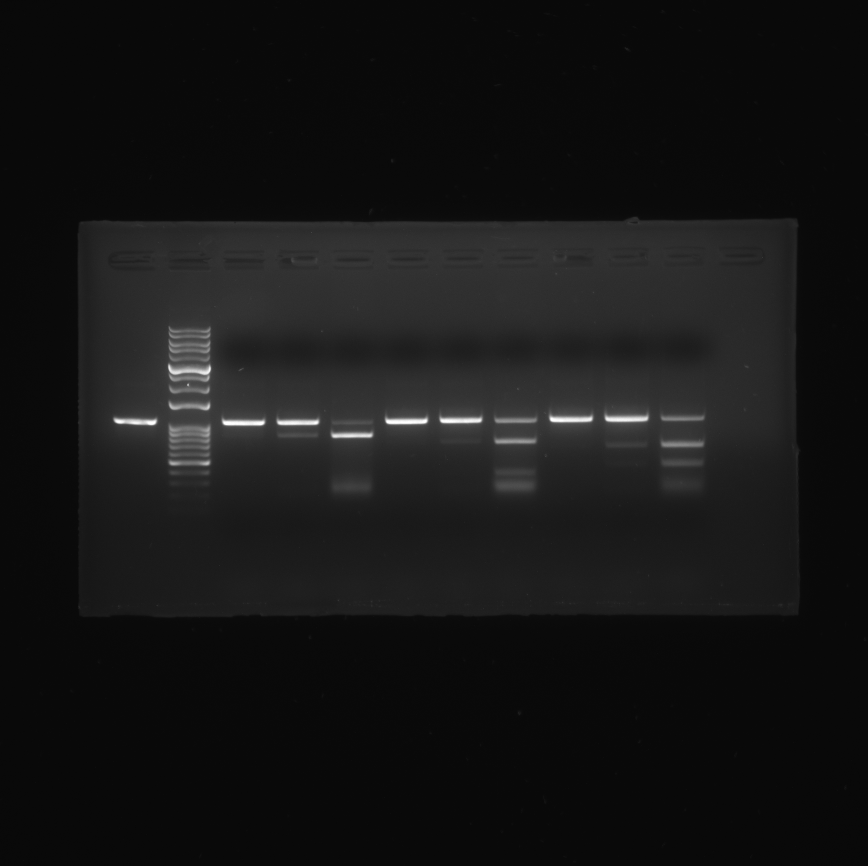

Supplement: Supplementary file 4 — Supplementary Information 1. [file 41598_2023_38331_MOESM4_ESM.tif]

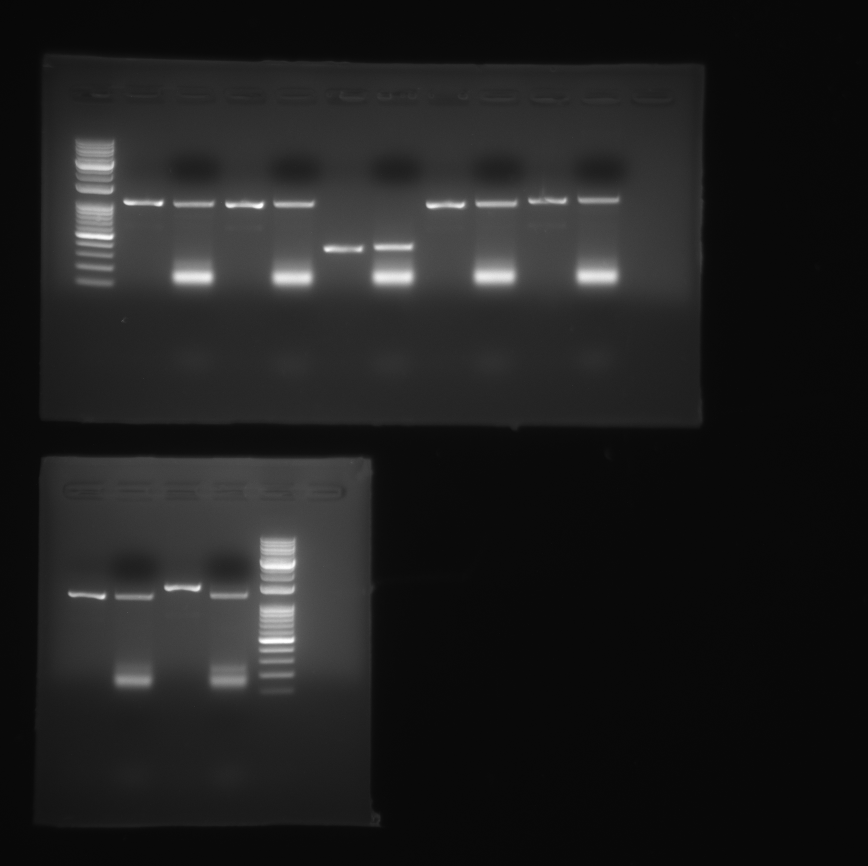

Supplement: Supplementary file 5 — Supplementary Information 2. [file 41598_2023_38331_MOESM5_ESM.tif]

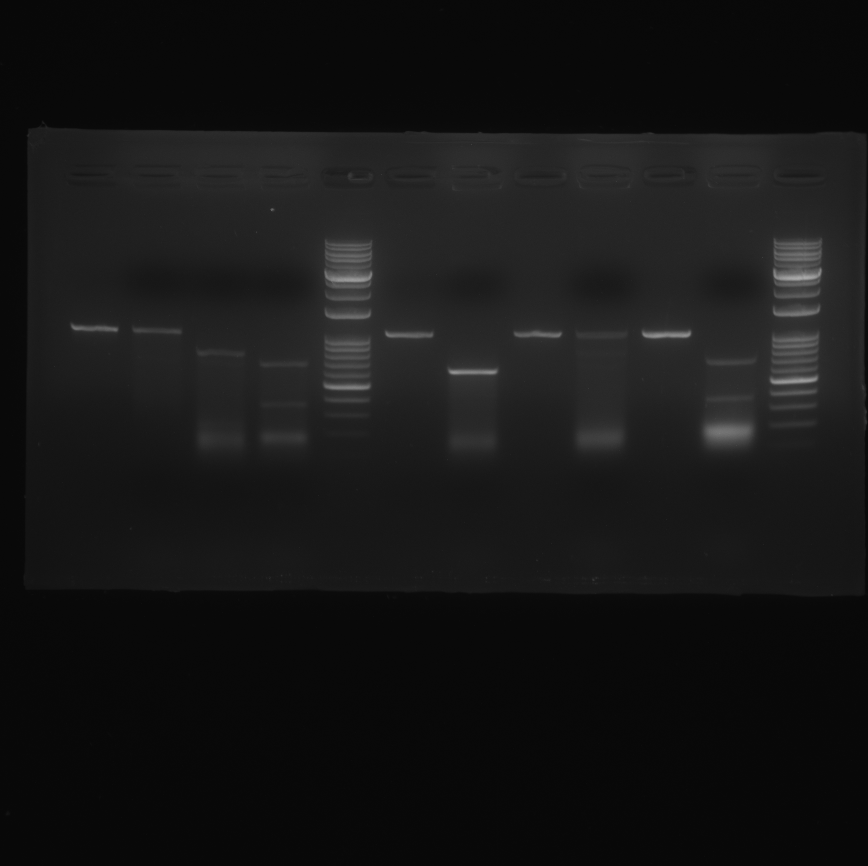

Supplement: Supplementary file 6 — Supplementary Information 3. [file 41598_2023_38331_MOESM6_ESM.tif]
