# Supplementary material for: The Cas9-gRNA ribonucleoprotein complex-mediated editing of pyrG in Ganoderma lucidum and unexpected insertion of contaminated DNA fragments
Source: Sci Rep. 2023 Jul 10;13:11133. doi: 10.1038/s41598-023-38331-2 (PMC10333205; doi:10.1038/s41598-023-38331-2)
Supplement: Supplementary file 7 — Supplementary Tables. [file 41598_2023_38331_MOESM7_ESM.docx]

**Supplementary Table S1. Primer used in this study**

| Name | Direction | Nucleotide sequence (5’ to 3’)^a^ |
| --- | --- | --- |
| gRNA | gRNA1_F | TAATACGACTCACTATAGGTTCAGGATTGTGGACATCGTGTTTTAGAGCTAGAAATAGCAAG |
|  | gRNA2_F | TAATACGACTCACTATAGGTCCGCGATCGAACGCCTCAAGTTTTAGAGCTAGAAATAGCAAG |
|  | gRNA3_F | TAATACGACTCACTATAGGGACAGAAAATTTGCCGACATGTTTTAGAGCTAGAAATAGCAAG |
|  | gRNA_R | AAAAAAGCACCGACTCG |
| *pyrG* | pyrG_F | GCAAAACTGGAAAACAGCGCG |
|  | pyrG_R | ACTGTGCTACTATACCCGTCTC |

^a^The crRNA sequences are underlined.

**Supplementary Table S2. Inserted sequences**

| **Plasmid DNA** | |
| --- | --- |
| N4 | ttcaggattgtggacattggcttaactatgcggcatcagagcagattgtactgagagtgcaccatcgt |
| N27 | ttcaggattgtggaccgaggcagcagatcaattcgcgcgcgaaggcgaagcggcatgcatttacgttgacaccatcgaatggtgcaaaacctttcgcggtatggcatgatagcgcccggaagagagtcaattcagggtggtgaatgtgaaaccgt |
| N29 | ttcaggattgtggacatgagaggcggtttgcgtattgggtgccagggtggtttttcttttcaccagtgagacgggcaacagctgattgcacttcaccgcctggccccgt |
| N37 | ttcaggattgtggacatcccctgacgggcttgtctgctcccggcatccgcttacagacaagctgtgaccgtctccgggagctgcatgcgt |
| N40 | ttcaggattgtggacatccacgatgttgccgaagatggggtggcgctcgtgcttcttgtcctcctccaccaggaagctctcctccaggcggtggaagaagctgtcgtccaccttggcccgt |
| N61 | **g**tcaggcggtatgggcaggttttcgtcgcccgcggggtaatctggtggcccagtcgctggcggcgcgggccatctcgatcacgatgttctcgggcttgtggcggcccatcaccttcaccagctcgtcca (E. coli chromosomal DNA in blue color) |
| ***G. lucidum* mtDNA** | |
| N10 | ttcaggattgtggacatttaacccatgtaaacctgttgaagcatagaatgtagtcccaaatactgagtctgttatagtaaatgtagcattaaaatattcgt |
| N11 | ttcaggattgtggacatttaacccatgtaaacctgttgaagcatagaatgtagtcccaaatactgagtctgttatagtaaatgtagcattaaaatattcgt |
| N22 | ttcaggattgtggacatactgaacttaaaggtttattttttaataatccattattaagtttaagtttaactatttgtttatttagtatggctggagtacctcctttattaggtttcttttcaaaacaatttgtattatattcggcaatgcaaagtgaatattactttattcgt |
| N26 | ttcaggattgtggacctcatatgaggtgggagtatatattttatatactaatattgcgatgtcgactcaacctatcctccgggggtagaagcttggaagggttcggctgttcgccgattaaaaggttacgccgt |
| N39 | ttcaggattgtggacattagggaatatacctaaaataatagtaggaatcaataaagctattaataaattaaattctaatctagttatatcttgcgt |
| N53 | ttcaggattgtggacattaattaagctaggtttagagcaactcattacaataaaactaaaaaattcgt |
| N34 | ttcaggattgtggacattaacccctacaaataatgtccagaagtgaatttttcctaataattcattgtatgttaaacctattatttttggtgtccagaaatagaacccagcaaataaagcaaatacagctcccattgataatacataatggaacgt |
| N66 | ttcaggattgtggacattaacccctacaaataatgtccagaagtgaatttttcctaataattcattgtatgttaaacctattatttttggtgtccagaaatagaacccagcaaataaagcaaatacagctcccattgataatacataatggaacgt |
| N74 | ttcaggattgtggacattaacccctacaaataatgtccagaagtgaatttttcctaataattcattgtatgttaaacctattatttttggtgtccagaaatagaacccagcaaataaagcaaatacagctcccattgataatacataatggaacgt |
| ***E. coli* chromosomal DNA** | |
| N36 | ttcaggattgtgtcggcacaaagcgtttaccggcaaagaagccaagatactcaggcagcttaatacggtagaaacggttaaacatgtacgctgcgatcgcaccggagataatccctccgag----------------------------------cgt |
| N42 | ttcaggattgtggactattctgggtcctacaatcaccgagcgagggtttctcaccgtcgaccgtatcgggaaactcgtatggttctgaggaagaacacgaagccccacgtcttcccctgtgagctcgacgtcgcctcgtccctcactcgccagagaggatcgttccttgctcccgcgggacgtccagctcgaccccgcgcgcgtcagcccagggtgcatgtgccgtacgtgcttctccctcagcgcgtccaaaagcgccgccgcgtccgcgcggacgtcgtccacgttgagcctgtaccgcaatctcttcaaaagtgatggttttgtgcttcaaccaacgttccgcgaggctgtccatatggccactacgccacgaaagtaactccttgacttcggactcgcctgtaccatgcccacgttgcgggt |
| N51 | ttcaggattgtggacatggtgttggtgcggaatactggcgtgattatttgaaactgagcgccaatggtcgt |
| N54 | ttcaggattgtggacattggttgccgaatgcggcgtaaacgccttatccggcccaggttttgctattaccaccagatttccatctgggcgt |
| N55 | ttcaggattgtggacatgccgccggagttgccctgaacgtttttaacccgcttcagactgccgagctgcaaaaatgcacagtagcatcgt |
| N56 | ttcaggattgtggacattatgggtcaacgatgataaaccgcaggtgattgtggtcggtttcgggcgttttgggcaggtgattggtcgtttgctgatggcaaataaaatgcgcattaccgtgctggagcgggatatcagcgccgttaacctgatgcgcaaatatggctacaaagtttattacggcgacgccacgcaggtagatcttttacgttctgcgggtgcagaggccgctgagtctatcgtcattacctgtaacgagccggaagacaccatgaagctggtggaaatatgccagcagcactttccgcatttgcatattcttgcgcgagcgctgtcggcgcaaaaaacattatccagaacgggagtgcgccttgagcgacacgaattatgcagtgatttacgacctgcacagccataccacagcttccgatggctgccgt |
| N60 | ttcaggattgtggacatggtgatgggcggattaattatcgcggctgtcgcccttgaccgtcttatcagtaagtaaggaattgaacatgaaaatctccccccgt |
| N65 | ttcaggattgtggacatgaccagaccttcttgatgatgggcaccggaagtggcgcgaaacaggtaaacggcgagcattctacccgtggaaataaaaaattaacagttgcgatttttcccctggcaggctttttctgcgccgtcgttgccggagcacaatggaacatctggtggcttgctgttgtaacgcagcattccaggcatcgcccagatacgcggtgacaaatttcttctctttcttgccatccgggcaggccatcatcgcgt |
| gRNA3 | gacagaaaatttgccgaaggcatgataaaacatcatcaacaaagatgaaacaagacgtaatatgttttgagtgaatgtaataaaaatagtgctgcaaaacaaaacgataccgcgatactctaagcttggataaatatgtgagatcaataaatgaaaattatcagttttgttctgccttgcttgctggtcctg cattggtaggatgtagtc |
| **Single base insertion** | |
| N23 | ttcaggattgtggacatgcgt |
